# Increased Sodium Concentration in Substantia Nigra in Early Parkinson's Disease: A Preliminary Study With Ultra-High Field (7T) MRI

**DOI:** 10.3389/fneur.2021.715618

**Published:** 2021-09-09

**Authors:** Stephan Grimaldi, Mohamed Mounir El Mendili, Wafaa Zaaraoui, Jean-Philippe Ranjeva, Jean-Philippe Azulay, Alexandre Eusebio, Maxime Guye

**Affiliations:** ^1^APHM, Hôpital Universitaire Timone, Department of Neurology and Movement Disorders, Marseille, France; ^2^APHM, Hôpital Universitaire Timone, CEMEREM, Marseille, France; ^3^Aix Marseille Univ, CRMBM, CNRS, Marseille, France; ^4^Aix Marseille Univ, CNRS, Institut de Neurosciences de la Timone, Marseille, France

**Keywords:** Parkinson's disease, neurodegeneration, sodium, biomarker, ultra high field magnetic resonance imaging

## Abstract

Pathophysiology of idiopathic Parkinson's disease (iPD) is complex and still misunderstood. At a time when treatments with disease-modifying potential are being developed, identification of early markers of neurodegeneration is essential. Intracerebral sodium accumulation could be one of them. Indeed, it may be in relation to the mitochondrial dysfunction that early exists in iPD. For the first time, we used brain sodium (^23^Na) MRI to explore sodium concentration changes that have already been reported to be related to neurodegeneration in other diseases. We prospectively included 10 iPD patients (mean age 52.2 ± 5.9 years-old) with motor symptoms that started <36 months before inclusion and 12 healthy subjects (mean age 53 ± 6.4 years-old). Patients were scanned in OFF medication state by using proton (^1^H) and ^23^Na MRI at 7T. We then extracted quantitative Total Sodium Concentration (TSC) from five regions of interest known to be early impaired in iPD [substantia nigra (SN), putamen, caudate nucleus, pallidum, thalamus] and in one region supposed to be relatively spared in the first stages of the disease [cortical gray matter (neocortex)]. Potential atrophy in these structures was also investigated with ^1^H MRI. Relative to healthy subjects, iPD patients showed higher TSC in the SN (43.73 ± 4.64 vs. 37.72 ± 5.62, *p* = 0.006 after Bonferroni correction). A trend of increase in sodium concentrations was found within the pallidum (45.80 ± 4.19 vs. 41.07 ± 4.94, *p* = 0.017), putamen (48.65 ± 4.58 vs. 43.66 ± 5.04, *p* = 0.041) and the cortical gray matter (56.34 ± 3.92 vs. 50.81 ± 5.50, *p* = 0.021). No significant brain atrophy was found in patients compared to controls. Thus, alteration of sodium homeostasis in the SN in the absence of atrophy could be considered as a potential early marker of cellular dysfunction in iPD.

## Introduction

Idiopathic Parkinson's disease (iPD) is a neurodegenerative disease affecting the central nervous system and is a major cause of disability and dependence. By 2030, the number of parkinsonian patients could increase by 56%, with 1 in 120 people over the age of 45 suffering from the disease (1). To date, there is no treatment to slow or stop the progression of the disease. At a time when treatments with disease-modifying potential are being developed (for example anti-synuclein antibodies or iron chelators) (2), identification of early markers of neurodegeneration is essential and these are urgently lacking.

Although α-synuclein misfolding is part of a significant upstream pathway leading to dopaminergic degeneration (3, 4), mitochondrial dysfunction is another main upstream pathway to parkinsonism (5). Among the deleterious consequences of mitochondrial dysfunction, increased intracellular sodium (Na^+^) concentration has been reported (6) and reflects an alteration of the cellular homeostasis. Interestingly, Na^+^ concentration changes in the brain have already been related to neurodegeneration, for example in Huntington's Disease (7) or Amyotrophic Lateral Sclerosis (8) with brain sodium (^23^Na) MRI which allows the exploration of sodium distribution through a non-invasive procedure. Thus, we hypothesize that sodium accumulation could be a potential early biomarker of iPD, especially in the substantia nigra (SN) where impairment of regulation of mitochondrial DNA copy number occurs and is thought to be related to neurodegeneration in iPD (9). This pathophysiological approach could lead to the identification of new therapeutic targets such as the Na^+^ K^+^ Cl^−^ cotransporter isoform 1 (NKCC1) importer antagonist bumetanide, which has been reported to attenuate motor effects of dopamine deprivation (10).

In this exploratory study, we propose to study brain sodium concentrations using ultra-high field (7T) MRI in iPD patients relative to healthy subjects.

## Materials and Methods

### Population Studied

We prospectively recruited 10 patients with iPD between February and November 2020. All patients met the iPD MDS diagnostic criteria (11) with motor symptoms that started <36 months before inclusion.

Twelve age- and gender-matched healthy subjects were also recruited prospectively during the same period.

Written informed consent was obtained from all participants. The study was approved by the local Ethics Committee (Comité de Protection des Personnes Sud Méditerranée 1), in accordance with the Declaration of Helsinki.

### Clinical Data Collection

All patients had been examined in a standardized manner within a half-day by a movement disorders specialist working in the Department of Neurology and Movement Disorders, Marseille University Hospital. We collected scores using different scales to evaluate the severity of symptoms [i.e., UPDRS III, Schwab & England, Hoehn & Yahr, SCOPA-AUT and REM-sleep behavior disorders (RBD) Screening Questionnaire (12)], cognition (MoCA, Lexical and semantic fluency, Benton Judgment of Line Orientation Test-15 items), mood disorder [Hospital Anxiety and Depression (HAD) scale] and apathy (Starkstein motivation scale). Levodopa equivalent dose was calculated (13). The clinical data are summarized in Table 1. After receiving the patient's agreement, dopaminergic treatment was suspended 72 h before scanning to evaluate the severity of the disease and levodopa responsiveness (good if >50%/bad). Indeed, the effect of exogenous dopaminergic intake on a possible modification of sodium concentrations and metabolism is unknown.

**Table 1:** Patients' characteristics.

	**Mean**	**SD**
Age (years)	55.2	5.9
Disease duration (months)	20.3	11.2
Schwab & England	97	4.8
Hoehn & Yahr	1	0
UPDRS III	12.9	3.8
MoCA	27.9	1.3
Total Lexical fluency (number in 2 min)	20.2	7.4
Total Semantic fluecny (number in 2 min)	28.8	3.9
Benton Test (15-items)	30	2.7
Starkstein motivation scale	19.9	5.7
HAD	11.8	5.9
SCOPA-AUT	9.7	7.8
RBD screen questionnaire	3.7	2.2
Levodopa (md/day)	80	188.9
Levodopa Equivalent Dose (mg/day)	175.5	259.2
	Number of subjects (%)	
Gender	8 M (80%) and 2 F (20%)	
Orthostatic hypotension	2 (20%)	
Dopamine agonist	4 (40%)	
Levodopa	3 (30%)	
IMAO-B	6 (60%)	
ICOMT	0 (0%)	
Anticholinergic	0 (0%)	
Levodopa Responsivness	Good for 10 (100%)	

*UPDRS, Unified Parkinson's Disease Rating Scale; MoCA, Montreal Cognitive Assessment; HAD, Hospital Anxiety and Depression scale; SCOPA-AUT, Scales for Outcomes in Parkinson's disease - AUTonomy; RBD, REM Sleep Behavior Disorder Screening Questionnaire; SD, standard deviation*.

### MRI Acquisition and Post-processing

MRI acquisition was performed with a 7-T Magnetom system (Siemens, Erlangen, Germany). For ^23^Na MRI exploration, we used a dual-tuned ^23^Na/^1^H QED birdcage coil and a multi-echo density adapted 3D projection reconstruction pulse sequence (TR = 120 ms, 5,000 spokes, 384 radial samples per spoke, 3 mm nominal isotropic resolution; to ensure a sufficient number and distribution of TEs, while taking into account the 5 ms readout of the sequence, we applied the sequence three times within the same exam in order to obtain 24 TEs ranging from 0.2 ms to 70.78 ms: 1st acquisition: 0.20 - 9.70 - 19.20 - 28.70 - 38.20 - 47.70 - 57.20 - 66.70 ms, 2nd acquisition: 1.56 - 11.06 - 20.56 - 30.06 - 39.56 - 49.06 - 58.56 - 68.06 ms, 3rd acquisition: 4.28 - 13.78 - 23.28 - 32.78 - 42.28 - 51.78 - 61.28 - 70.78 ms, total acquisition time = 3 × 10 min) adapted from (14). Six tubes with known sodium concentrations (from 25 to 100 mmol/L within 2% of agar gel) were placed within the field of view to serve as a reference for quantification (14). A 32-channel phased-array ^1^H head coil (1Tx/32 Rx; Nova) was used to acquire a sub-millimeter ^1^H three-dimensional Magnetization-Prepared 2 Rapid Acquisition Gradient-Echoes (MP2RAGE) sequence (TR = 5,000 ms/TE = 3 ms/TI1 = 900 ms/TI2 = 2,750 ms, 256 slices, 0.6 mm isotropic resolution, acquisition time = 10 min 12 s).

Sodium images were reconstructed offline, fitted using a bi-exponential model, and finally normalized relative to signal from reference tubes to obtain quantitative Total Sodium Concentration (TSC) maps of the whole brain. We followed the methodology previously described in (14). This multi-echo approach allows to account for the complex relaxation processes of ^23^Na through a bi-exponential fitting enabling to determine the ordinate at TE = 0 ms of the curve corresponding to the TSC. In the present preliminary study with limited sample size and in order to minimize the statistical type II error, we decided to focus the analysis solely on TSC, the metric mainly expressed in the literature. Increase in sample size will allow to evaluate relative variations of short and long T2^*^ pool fractions of ^23^Na. The first ^23^Na echo (TE = 0.20 ms) and the ^1^H images were coregistered using a rigid transformation (15). ^1^H images were segmented into Gray Matter (GM), White Matter (WM) and cerebrospinal fluid (CSF) (0.9 tissue probability threshold) using the Statistical Parametric Mapping 12 “New Segment” tool (16), into deep gray matter (DGM) using FSL-FIRST tool (accumbens, amygdala, caudate, hippocampus, pallidum, putamen and thalamus) (17) and into substantia nigra by registering the ^1^H images to the Montreal Neurologic Institute (MNI) 152 space and bringing back the SN-AAL3 mask to the subject space (15). without resectioning (SPM8; https://www.fil.ion.ucl.ac.uk/spm/software/spm8/). The ^1^H images were then segmented and normalized into the MNI template, and the resulting transformation was applied to the quantitative ^23^Na maps. Quantitative TSC values were extracted from five regions of interest known to be early impaired in iPD (SN, putamen, caudate nucleus, pallidum, thalamus) and in one region supposed to be relatively spared in the first stages of the disease, the cortical gray matter (neocortex). Cerebral volumes were normalized for head size using the intracranial volume (18).

### Statistical Analysis

Categorical variables are presented as numbers and percentages, and the quantitative results as a mean with standard deviation. Comparisons between groups were made with Chi2 or Fisher tests for categorical data and Wilcoxon for continuous data, as appropriate. Correlations between clinical features and MRI parameters were looked for with Spearman correlation analysis. A two-sided *p*-value < 0.05 was considered statistically significant in univariate analysis and *p* < 0.008 after Bonferroni correction. Statistical analyses were performed using JMP software JMP 9.0.1 (SAS Institute).

## Results

### Patients' and Healthy Subjects' Characteristics

Eight males and 2 females with iPD were included in this study with a mean age of 55.2 ± 5.9 years-old and a mean disease duration of 20.3 ± 11.2 months. All of them were completely independent in their daily life as shown by scores of Schwab & England (97 ± 4.8) and Hoehn & Yahr (1 ± 0). No cognitive impairment was identified (MoCA score at 27.9 ± 1.3). Mean UPDRS III OFF anti-parkinsonian drugs was 12.9 ± 3.8. Clinical data are summarized in Table 1.

iPD patients were comparable with healthy subjects concerning age (mean age of 53.0 ± 6.4, *p* = 0.35) and gender (6 F and 6 M, *p* = 0.20).

### Sodium Brain Accumulation and Brain Volume

**Figure 1:**
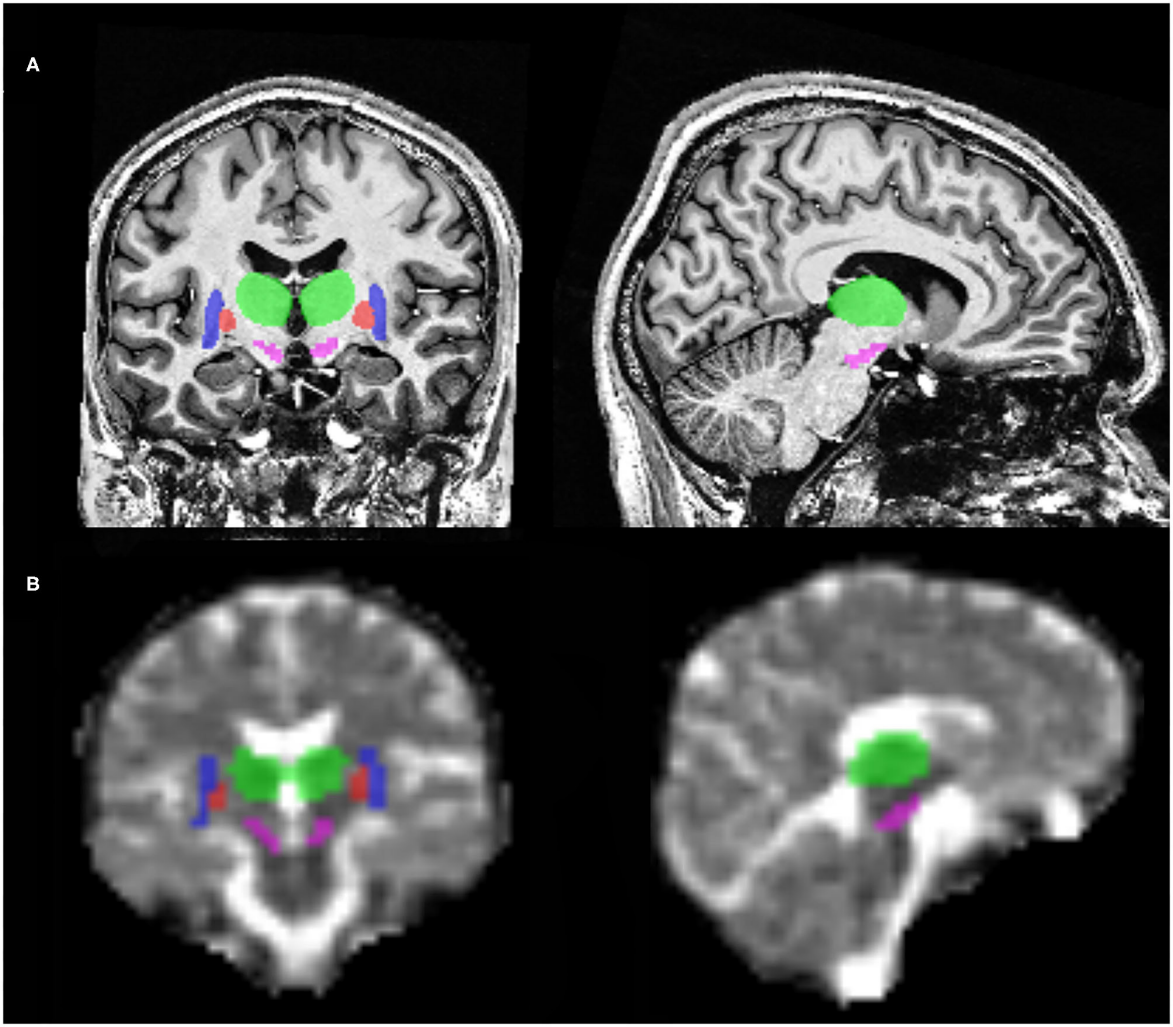
Illustration of regions of interest with coronal and sagittal brain MRI slices at 7T. **(A)** ^1^H T1-weighted MP2RAGE slices. **(B)** ^23^Na MRI slices. In this illustration, can be seen the substantia nigra (pink), putamen (blue), pallidum (red) and the thalamus (green).

**Figure 2:**
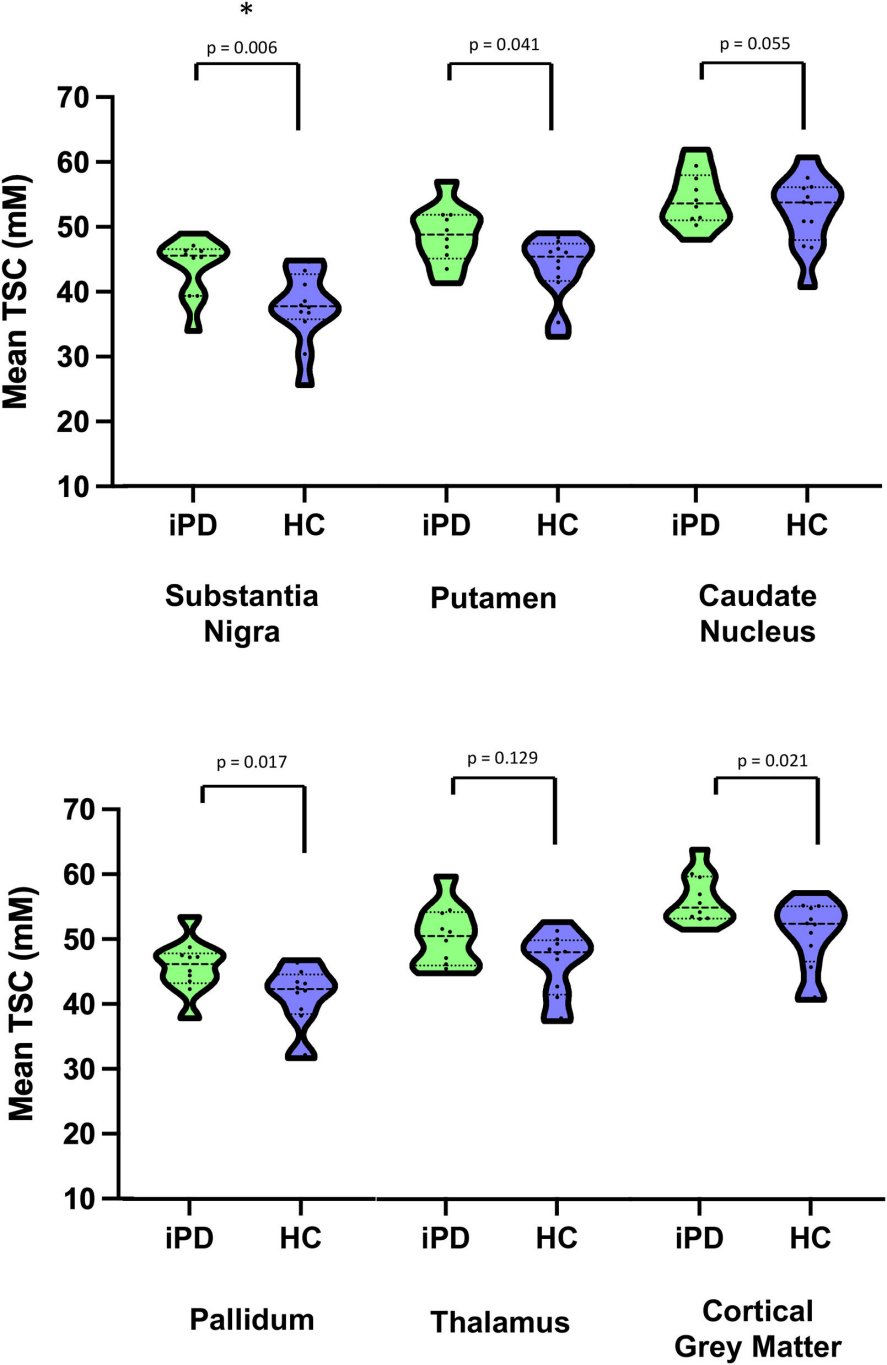
Violin plots of Mean Total sodium concentration (TSC) (wet tissue volume, mmol/l) for iPD and HC. *Survive after multiple comparisons. iPD, idiopathic Parkinson's Disease; HC, Healthy Controls; TSC, Total sodium concentration.

As shown in Table 2, Figures 1 and 2, relative to healthy subjects, patients with iPD showed higher TSC in the SN (43.73 ± 4.64 vs. 37.72 ± 5.62, *p* = 0.006 after Bonferroni correction). A trend of increase in sodium concentrations was found within the pallidum (45.80 ± 4.19 vs. 41.07 ± 4.94, *p* = 0.017), putamen (48.65 ± 4.58 vs. 43.66 ± 5.04, *p* = 0.041) and the cortical gray matter (56.34 ± 3.92 vs. 50.81 ± 5.50, *p* = 0.021).

**Table 2:** Mean Total sodium concentration (TSC) (wet tissue volume, mmol/l) and normalized volumes of brain regions of interest.

**Brain areas**	**Group**	**TSC ± SD**	***p***	**Normalized volume ± SD**	***p***
Substantia Nigra	iPD	43.73 ± 4.64	0.006^*^	0.07 ± 0.01	0.149
	Healthy subjects	37.72 ± 5.62		0.08 ± 0.01	
Putamen	iPD	48.65 ± 4.58	0.041	0.62 ± 0.14	0.187
	Healthy subjects	43.66 ± 5.04		0.69 ± 0.11	
Caudate nucleus	iPD	54.22 ± 4.36	0.055	0.48 ± 0.07	0.553
	Healthy subjects	52.35 ± 5.50		0.50 ± 0.07	
Pallidum	iPD	45.80 ± 4.19	0.017	0.23 ± 0.03	0.461
	Healthy subjects	41.07 ± 4.94		0.24 ± 0.04	
Thalamus	iPD	50.60 ± 5.92	0.129	0.97 ± 0.14	0.468
	Healthy subjects	46.29 ± 5.14		1.01 ± 0.15	
Cortical Gray Matter	iPD	56.34 ± 3.92	0.021	42.53 ± 5.67	0.714
	Healthy subjects	50.81 ± 5.50		43.37 ± 4.98	

* *Survive after multiple comparisons. TSC, Total sodium concentration; SD, standard deviation; iPD, idiopathic Parkinson's Disease*.

No significant difference in ROI normalized volumes was found between patients and controls.

There was no correlation between clinical features, levodopa equivalent doses and MRI parameters.

## Discussion

Despite the limited number of subjects included in this study, we have been able to demonstrate an accumulation of sodium in patients with iPD. This accumulation is significant in the SN at this early stage of the disease.

SN is recognized as the origin of the nigro-basal ganglia-thalamic-cortical motor pathway dysfunction in iPD. Indeed, the most consistent finding of the neuropathology of iPD is a loss of dopaminergic neurons in the SN pars compacta which modulates activity of neurons in the basal ganglia. In the end, inadequate facilitation of the corticospinal tracts produces akinesia and bradykinesia which are part of parkinsonian syndrome (19). Pathophysiology of iPD is still misunderstood but along with α-synuclein misfolding, mitochondrial dysfunction is considered to have an important role in neurodegeneration (5). Changes in mitochondrial gene expression, well-documented in the SN (9), seem to contribute to cellular energy failure, which in turn leads to loss of function of Na^+^/K^+^ ATPase and impaired ability of the cell to maintain resting potential and to export Na^+^ (6). Then, the significant sodium accumulation in the SN is believed to be a sign of early dopaminergic neurons dysfunction in iPD. Also, the trend of increase in sodium concentrations within the pallidum corroborates the early SN-basal ganglia neural circuits impairment in iPD but more investigations are warranted to support these results.

The trend of sodium accumulation in cortical gray matter was not fully expected at this stage, in the absence of obvious cognitive impairment. However, early changes in personality and mild cognitive impairment have been described in iPD (20). Characteristically, these changes are often subtle at the beginning and difficult to detect without specific neuropsychological tests. Deficits mainly affect executive function including working memory and visuospatial capacity. Here, 3 patients had a MoCA score at 26/30 which is the limit for normal values (21). Lewy pathology, hallmark of iPD, in the cerebral cortex does not correlate with cognitive impairment. Nevertheless, recent studies have shown abnormal mitochondria content and function, and increased oxidative stress and oxidative responses in the frontal cortex in iPD (22) that could be related to the tendency of sodium accumulation associated with early cortical dysfunction.

A variety of consequences of Na^+^ accumulation may be relevant in PD pathophysiology. Mitochondrial dysfunction, ionic disturbances and neurodegeneration are interrelated biological processes already described iPD (5). Mitochondrial dysfunction may result in intraneuronal sodium overload through reversed activity of the sodium-calcium exchanger and axonal calcium import (6). In turn, this calcium overload may force the opening of mitochondrial permeability transition pore, leading to retrograde electron flux through the electron transport chain, resulting in increased reactive oxygen species production, release of cytochrome c, and activation of apoptosis (23, 24) particularly in SN dopaminergic neurons which project their axons on basal ganglia nuclei (25). We hypothesize that this accumulation of Na^+^ may affect the activity of other structures in the SN-basal ganglia-thalamic-cortical circuit. For example, sodium ions are essential for spike generation in midbrain dopaminergic neurons (26) and pallidum neurons (27). A computational modeling study recently demonstrated a reduced availability of sub-threshold activated sodium and potassium channels, resulting in a decrease in firing regularity in pallidum neurons (28). Also, dopamine transporter (DAT) is a sodium-coupled transmembrane protein that mediates the reuptake of dopamine from the synaptic cleft, and is localized to presynaptic nigrostriatal terminals (29). It operates by coupling transport of Na^+^ along its concentration gradient from the extracellular compartment to the intracellular compartment allowing the transport of the substrate in the same way (30). Intracellular Na^+^ overload might deteriorate its functioning and worsen neurological symptoms.

From a therapeutic point of view, interestingly, it has been shown that the neuronal Na^+^ K^+^ Cl^−^ cotransporter isoform 1 (NKCC1) importer antagonist bumetanide, which reduces intracellular Cl^−^ levels (but also reduces Na^+^ and K^+^ intracellular import) attenuates motor effects of dopamine deprivation by restoring GABAergic inhibition including the cortico-striatal pause-rebound response (31). Damier et al. (10), reported an improvement of iPD motor symptoms in the 4 patients treated with bumetanide which also improved gait and freezing in 2 of these patients calling for double-blind, placebo-controlled, randomized trials to confirm the therapeutic efficacy of bumetanide. NKCC1 importer antagonist has been postulated to have also a neuroprotective effect in iPD through its action on astrocytes, microglia and oligodendrocytes by limiting the intracellular accumulation of NA^+^, Cl^−^ and K^+^ (32).

We did not find any significant atrophy when we compared the volumes of brain structures in iPD patients and healthy subjects although sodium concentration was abnormally elevated in patients. This suggests that abnormal elevated sodium concentration more likely reflects cellular dysfunction rather than cell death. This finding is in favor of the hypothesis that elevated sodium concentration could be a potential marker of early processes of neurodegeneration before neuronal death.

Our study has some limitations. The main one is the limited number of participants. This preliminary study has therefore allowed us to demonstrate the feasibility and relevance of assessing brain sodium accumulation in iPD. Unfortunately, several structures implicated in iPD pathophysiology are too small to be evaluated individually in sodium imaging such as locus coerulus, subthalamic-nucleus or SN pars compacta and SN pars reticulata separately due to the limited spatial resolution of sodium MRI even at high field.

## Conclusion

To our knowledge, this is the first study using sodium MRI at 7T providing evidence of abnormal sodium concentration in SN which is a region known to be particularly impaired early in iPD. The evidence of such an alteration of sodium homeostasis in the absence of atrophy could be considered as a potential marker of early processes of cellular dysfunction and neurodegeneration before neuronal death in iPD. Further investigations are needed to confirm these results and to explore mapping of sodium homeostasis that might be different in other parkinsonian syndromes.

## Data Availability Statement

The raw data supporting the conclusions of this article will be made available by the authors, without undue reservation.

## Ethics Statement

The studies involving human participants were reviewed and approved by Comité de Protection des Personnes Sud Méditerranée 1. The patients/participants provided their written informed consent to participate in this study.

## Author Contributions

SG: conception and design of the study, acquisition and analysis of data, drafting the manuscript and figures, and approval of final version of submitted manuscript. ME-M and J-PR: analysis of data, drafting the manuscript, approval of final version of submitted manuscript. WZ: analysis of data, drafting the manuscript, drafting the figure, approval of final version of submitted manuscript. J-PA, AE, and MG: conception and design of the study, drafting the manuscript, and approval of final version of submitted manuscript. All authors contributed to the article and approved the submitted version.

## Funding

This work was supported by ANR (ANR-15-CE19-0019-01, NEUROintraSOD-7T) and A^*^MIDEX (A^*^MIDEX-EI-17-29-170228-09.43-Imetionic-7). Also, this work was performed by a laboratory member of France Life Imaging network (grant ANR-11-INBS-0006) and on the platform 7T-AMI, a French Investissements dAvenir programme (grant ANR-11-EQPX-0001).

## Conflict of Interest

The authors declare that the research was conducted in the absence of any commercial or financial relationships that could be construed as a potential conflict of interest.

## Publisher's Note

All claims expressed in this article are solely those of the authors and do not necessarily represent those of their affiliated organizations, or those of the publisher, the editors and the reviewers. Any product that may be evaluated in this article, or claim that may be made by its manufacturer, is not guaranteed or endorsed by the publisher.
